# Preprocessing on the Go: Practices in Gait‐Related Mobile EEG

**DOI:** 10.1111/psyp.70352

**Published:** 2026-06-25

**Authors:** Vaishali Vinod, Lara Johanna Papin, Robbin Romijnders, Walter Maetzler, Julius Welzel

**Affiliations:** ^1^ Department of Neurology University Hospital Schleswig‐Holstein Campus Kiel and Kiel University Kiel Germany; ^2^ Neuropsychology Lab, Department of Psychology Carl von Ossietzky University of Oldenburg Oldenburg Germany

**Keywords:** artifact rejection, gait, mobile EEG, preprocessing, walking

## Abstract

Mobile EEG has become popular in investigating brain dynamics during gait in recent years. Within this development, new preprocessing pipelines have been introduced and refined. The diversity of approaches, however, complicates comparisons across studies. To provide clarity, we reviewed studies that combined mobile EEG with gait measurements to map the preprocessing pipelines used in the field. Our review identified substantial heterogeneity in pipeline steps, their order, combinations, and the level of reporting detail. We visualized this heterogeneity as a map, tracing pathways from raw data to outcomes such as Power spectral density (PSD), Event‐related spectral perturbations (ERSP), Event‐related (de‐) synchronization (ERD/ERS), and Corticomuscular coherence (CMC), along with a subsequent analysis highlighting unique pipelines. Notably, artifact rejection varied across studies in both the tools used and reporting practices. While differences in hardware, setup, and experimental paradigms can justify this variability, they also challenge comparability across findings. These results emphasize the need for transparent reporting standards and provide a foundation for future efforts toward developing shared standards in the mobile EEG community.

## Impact Statement

Despite rapid growth in mobile EEG studies of gait, preprocessing practices remain highly diverse and inconsistently reported. By systematically mapping preprocessing pipelines across gait paradigms, cohorts, and neural outcome measures, this review provides the first structured overview of how preprocessing choices and their ordering vary across the field.

## 1 Introduction

Mobile electroencephalography (EEG) has experienced rapid growth in cognitive neuroscience over the past decade, enabling the study of brain activity not only during constrained laboratory tasks, but also during naturalistic movements. Among its applications, the study of gait has become a major focus. Mobile EEG during walking allows direct recording of cortical activity underlying locomotion (Richer et al. 2024; Song and Nordin 2021). Although walking is often considered to rely primarily on automatic subcortical and spinal processes under stable conditions, sensorimotor and associative cortical areas remain active even during steady, unchallenged walking (Delval et al. 2020). Cortical engagement further increases when walking requires sensory integration, attention, or environmental adaptations (Gwin et al. 2011; Petersen et al. 2012; Richer et al. 2024; Song and Nordin 2021). This makes the neural basis of gait not only of theoretical interest but also clinically relevant, particularly in conditions that can show gait impairments such as Parkinson's disease (PD), stroke, and age‐related mobility decline.

To better put the mobile EEG recordings into context, they have been recorded alongside other modalities, such as optical motion capture (OMC) and inertial measurement units (IMUs). These types of motion recordings can be additionally combined with electromyography (EMG) to link cortical signals with muscle activity and movement dynamics. The experimental context, including cohort characteristics, gait tasks, electrode type, and measurement system, often strongly influences both the neural signals recorded and the preprocessing approaches required. For instance, treadmill versus overground gait, or clinical versus healthy cohorts, can bring differences in signal quality, artifact patterns, and the interpretation of cortical dynamics. Depending on the research question and the setup, a recent review highlighted four outcome measures for characterizing cortical dynamics during gait (Richer et al. 2024):

- Power spectral density (PSD): Quantifies oscillatory power across frequency bands, often used to assess changes in rhythmic activity during gait (Nordin et al. 2020; Stuart et al. 2021). For example, PSD modulations in premotor and parietal cortices are known to scale with task demands such as walking speed or treadmill incline (Bradford et al. 2016; Bruijn et al. 2015; Bulea et al. 2015).
- Event‐related spectral perturbations (ERSP): Aligns cortical oscillations to discrete gait events (e.g., heel strike), to capture time‐frequency dynamics within the step cycle. This approach reveals phase‐specific theta and beta modulations during tasks involving gait adaptation or perturbation (N. A. Jacobsen and Ferris 2024; Nordin et al. 2020; Peterson and Ferris 2018; Wagner et al. 2016).
- Event‐related (de)‐synchronization (ERD/ERS): Reflects transient decreases/increases in rhythmic power relative to a baseline, closely linked to motor preparation and execution. In the context of gait, beta ERD typically precedes swing initiation, while beta ERS aligns with limb contact and postural stabilization (Borhanazad et al. 2024; Bradford et al. 2016).
- Corticomuscular coherence (CMC): Measures the functional synchronization between EEG and EMG signals, indicative of supraspinal contributions to gait, particularly in distal muscles during swing (Jensen et al. 2019; Roeder et al. 2018, 2020; Winslow et al. 2016). Multiple studies report reduced CMC during walking in PD and older adults compared to young adults (Roeder et al. 2020; Yokoyama et al. 2020), and that dopaminergic medication can increase cortico‐muscular connectivity across gait phases (dos Santos et al. 2024).

Each of these outcome measures requires a specific preprocessing strategy tailored to the specific signals it aims to isolate. Although synchronization with gait events and filtering are fundamental steps, subsequent preprocessing requirements vary depending on the outcome measure of interest. For example, ERSP and ERD/ERS rely on precise event‐locking and accurate baseline correction to detect the transient time‐frequency modulations that occur within the gait cycle (Grandchamp and Delorme 2011; Hu et al. 2014). CMC relies on precise alignment between EEG and EMG, along with frequency‐domain filtering, to minimize cross‐talk while preserving corticomuscular coupling (Liu et al. 2019). Decisions about filters, artifact‐removal methods, and signal decompositions therefore have a direct impact on what can be recovered as a neural signal and how confidently it can be interpreted. Collectively, these outcome measures portray gait as a behavior shaped by dynamic cortical engagement, with deviations reflecting age, disease, or therapeutic intervention (Mustile et al. 2023; Possti et al. 2021; Wang et al. 2020).

Analyzing gait‐related mobile EEG activity presents unique methodological challenges. EEG recorded during locomotion is susceptible to a complex mixture of physiological (ocular, muscle activity) and non‐physiological (electrode shifts, cable sway) artifacts (Castermans et al. 2014; Kline et al. 2015; Richer et al. 2024). These artifact profiles differ significantly by mobility task and hardware configuration. The term “mobile EEG” itself encompasses a wide range of paradigms, ranging from overground walking in natural environments to controlled treadmill tasks in a laboratory (N. A. Jacobsen and Ferris 2023; Nenna et al. 2021; Roeder et al. 2018; Winslow et al. 2016). To provide a standardized framework for comparing studies, Bateson and colleagues introduced a categorization of mobile EEG (CoME) scheme, which scores studies based on device, participant, and setup characteristics (Bateson et al. 2017). While this offers a practical way to account for differences in mobility contexts, it does not provide a framework for describing the spectrum of artifacts that can arise in mobile EEG recordings in each scenario, depending on individual gait patterns and environmental complexity.

Recent improvements in mobile EEG hardware have made it possible to measure brain activity during movement. Compact, head‐mounted amplifiers placed close to the electrodes have been shown to reduce cable movement and associated motion artifacts, while improving portability (De Vos and Debener 2014; Debener et al. 2012). Integration of EMG, eye tracking, and motion sensors into mobile EEG setups further supports artifact identification (Gramann et al. 2011; Niso et al. 2023). However, mobile recordings remain highly vulnerable to motion‐ and muscle‐related artifacts. Importantly, hardware characteristics such as amplifier placement, cable length, and electrode configuration can directly influence the severity and structure of these artifacts, and therefore also the preprocessing strategies required. Controlled comparisons have demonstrated that amplifier placement alone can substantially affect signal quality during gait recordings, even when other acquisition parameters are held constant (Scanlon et al. 2021). These challenges have driven the development of dedicated preprocessing toolboxes and pipelines for mobile EEG, including general‐purpose EEG environments (e.g., EEGLAB‐based workflows) and frameworks that integrate motion data and multimodal synchronization. Although these tools differ in how they implement filtering, artifact attenuation, and signal decomposition, all require user‐defined parameters and involve trade‐offs between noise reduction and signal preservation. As a result, differences in implementation and parameter choices can limit the comparison of resulting neural outcomes across studies using different pipelines. Automated tools, such as the BeMoBIL pipeline, provide structured workflows for multimodal data synchronization and cleaning (Klug et al. 2022). However, no single preprocessing approach can accommodate all mobile EEG systems or analytical outcomes. The central challenge is achieving an appropriate balance between suppressing motion‐related artifacts and preserving signals of interest. This balance depends on the level of motion contamination, the gait task, the cohort, and the neural outcome being targeted, whether PSD, ERSP, ERD/ERS, or CMC. Consequently, researchers tailor thresholds and preprocessing choices to their specific data and hardware, resulting in variation in preprocessing practices across studies.

ICA decomposes EEG into brain and non‐brain components. Non‐brain components, for example, eye movements and muscle activity, are generally considered as artifacts to be attenuated. However, the use of the remaining brain components differs across studies. In one approach, all non‐brain components are removed, and the cleaned signal is back‐projected to channel space for subsequent analysis (Jung et al. 2000; Chaumon et al. 2015). In the other, specific brain components are identified and studied directly at the source level, meaning that not all brain components are necessarily of interest for a given research question (Delorme and Makeig 2004; Gramann et al. 2011). However, in practice, this separation is not always clean. Individual components can contain mixtures of brain and non‐brain activity, making their classification ambiguous and the decision to retain or reject them dependent on the previously described approaches (Chaumon et al. 2015). In gait‐related EEG, this distinction is particularly challenging because locomotion itself generates physiological activity, including muscle activation, eye movements, and gait‐synchronized dynamics, some of which may be behaviorally meaningful rather than purely artifactual.

To attenuate artifacts in mobile EEG recordings, researchers use a mix of manual and automated approaches. Manual inspection and hand‐marking noisy channels are common, but are subjective and time‐consuming. Automated methods, such as artifact subspace reconstruction (ASR), target high‐amplitude, non‐stationary artifacts (Chang et al. 2018; Delorme and Makeig 2004; Mullen et al. 2013), while decomposition techniques, like independent component analysis (ICA) and canonical correlation analysis (CCA), help separate artifactual from neural sources (Kline et al. 2015; Klug et al. 2021; Snyder et al. 2015). These techniques are essential, yet each comes with constraints. Aggressive ASR thresholds or the removal of too many ICA components can inadvertently eliminate neural activity alongside artifacts (Artoni et al. 2017; Nathan et al. 2016). This is relevant for gait‐related mobile EEG, as locomotion has been associated not only with low‐frequency modulations but also with beta‐ and gamma‐band activity linked to motor control and sensorimotor integration (Cheng et al. 2025; Kimoto et al. 2024). Overly aggressive cleaning may therefore reduce or distort physiologically meaningful movement‐related signals (Klug et al. 2021, 2024). Determining whether preprocessing is overly aggressive requires explicit definitions of both artifact‐related signals and neural features of interest. Under these conditions, preprocessing performance can be evaluated in terms of sensitivity (extent of artifact attenuation) and specificity (preservation of neural signal), as proposed in recent work (N. S. J. Jacobsen et al. 2022). However, such evaluations are rarely implemented in practice, further contributing to uncertainty regarding the impact of preprocessing choices on downstream results.

For this reason, retaining as many components as feasible and explicitly reporting the number and type of rejected components is critical for interpreting and comparing findings across studies (Chaumon et al. 2015; Gonsisko et al. 2023). Another important consideration is that the performance of ICA depends on how well the data meet its underlying assumptions, including statistical independence of sources and relative stationarity of the signals. These assumptions can be influenced by preprocessing choices such as high‐pass filtering, as well as by the amount and quality of available data (Winkler et al. 2015). Importantly, deviations from these assumptions are not unique to mobile EEG and may also arise in stationary recordings depending on acquisition and preprocessing settings. Heterogeneity in preprocessing is therefore partly an expected consequence of working with diverse hardware and study designs.

The challenge arises when methodological diversity is poorly documented in studies that aim to quantify comparable neural outcomes. Without transparent reporting, it becomes difficult to determine whether differences in results reflect true physiological effects or arise from undocumented preprocessing choices, even in cases of similar tasks and outcome measures. Other neuroimaging fields have responded to similar issues through large‐scale standardization initiatives (Keil et al. 2014). Agreed Reporting Template for EEG Methodology—International standard (ARTEM‐IS) provides a machine‐readable template for structured EEG methodology reporting (Styles et al. 2021), while EEGManyPipelines (Trübutschek et al. 2024) and EEGManyLabs (Pavlov et al. 2021) quantify how different preprocessing decisions influence results rather than enforcing a single standard. Furthermore, in the fMRI community, fMRIPrep established a fully automated, consensus‐driven processing pipeline, built upon the Brain Imaging Data Structure (BIDS), to minimize subjective and biased decisions (Esteban et al. 2019). Collectively, these efforts highlight the importance of clarity and standardized documentation in evaluating and comparing scientific findings.

As mobile brain imaging (MoBI) expands toward more complex and multimodal paradigms (Klapprott et al. 2024; Nann et al. 2019; Robles et al. 2021), establishing similar transparency in mobile EEG preprocessing is especially important, given the lower signal‐to‐noise ratios. The present review provides a structured and descriptive analysis of preprocessing pipelines used in gait‐related mobile EEG research. By combining an automated literature review tool with a conventional database search, we identify common practices and highlight methodological divergences across gait‐related mobile EEG studies. Importantly, this work is not intended to evaluate pipeline quality or provide guidelines. Instead, it documents current practices as a foundation for future efforts toward systematic evaluation and standardization.

## 2 Methods

### 2.1 Literature Search

A two‐stage literature search was conducted to identify peer‐reviewed studies on gait and mobile EEG. Firstly, a custom Python script was used to query and download full‐text articles from the PubMed Central (PMC) Open Access subset in BioC format (Comeau et al. 2019). The search was conducted on May 15, 2025, using the search string: “(*mobile EEG*) AND (*gait*)”. To balance specificity and coverage, these keywords were automatically expanded in the script using Medical Subject Headings (MeSH), a controlled vocabulary in PubMed that indexes standardized synonyms and related concepts. This approach enabled a systematic and reproducible search. All PMC IDs retrieved per query were logged. All scripts are publicly available at: https://github.com/vaishalivinod/LitExtract.

Secondly, to extract relevant studies not indexed in the PMC Open Access repository, a manual search was performed on the PubMed database on June 16, 2025, using the same query. Review articles, protocols, duplicates, and dataset articles were excluded. We prioritized repositories with machine‐readable full text (PMC OpenAccess BioC) to enable reproducible extraction. The manual PubMed step used the same query to maintain consistency across sources.

### 2.2 Study Inclusion

**FIGURE 1:**
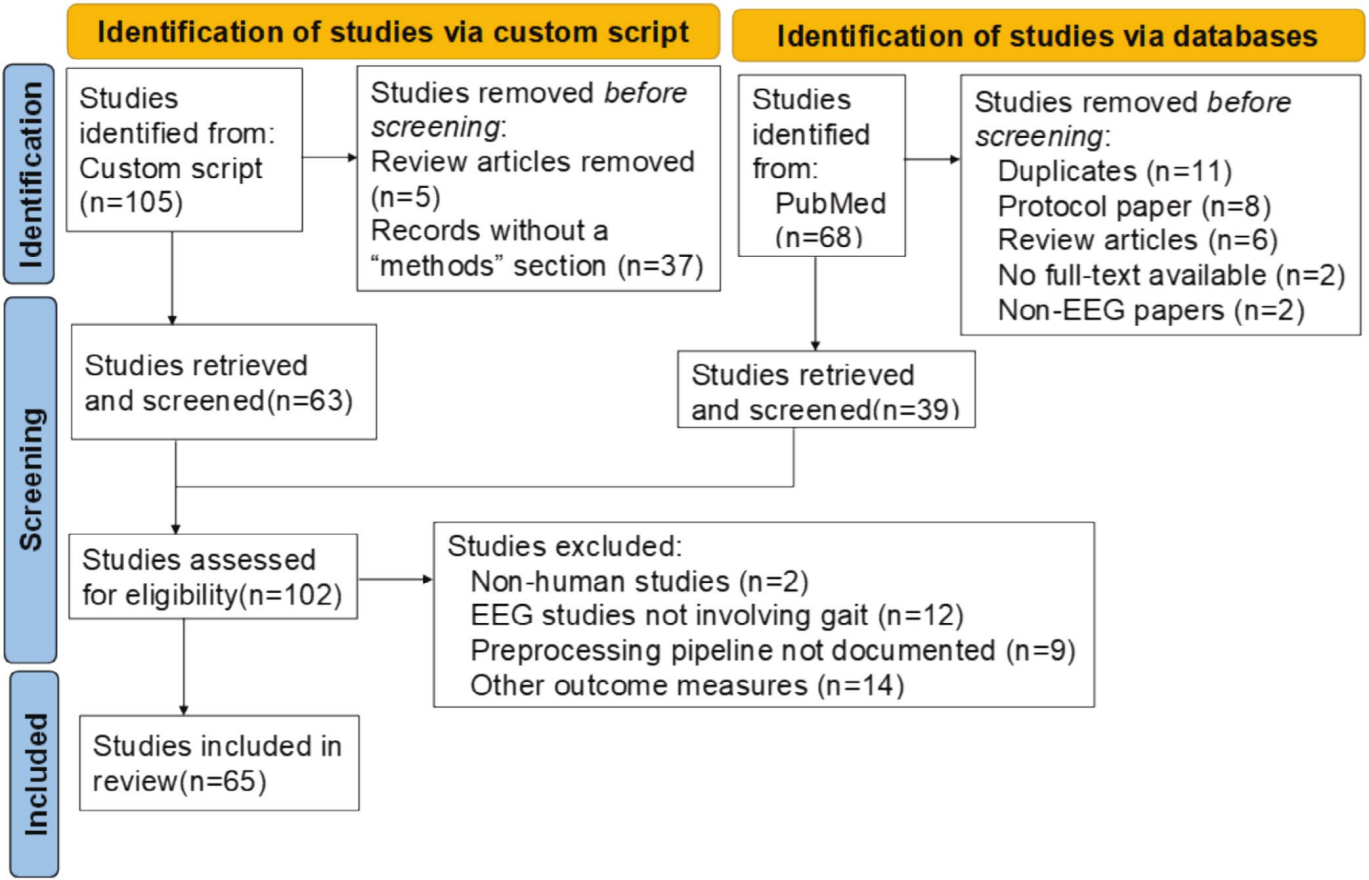
PRISMA flowchart of identification of studies with the automated retrieval and PubMed database.

Inclusion and exclusion criteria were defined prior to screening. Studies were eligible if they met all three criteria: (i) employed mobile EEG to record neural activity during walking (treadmill or overground), (ii) explicitly documented at least one preprocessing step within the EEG analysis pipeline, and (iii) peer‐reviewed research articles. Exclusion criteria filtered out animal studies, review articles, methodological protocols, duplicate publications, and dataset articles. Additionally, studies with a primary focus other than gait (e.g., Brain‐Computer Interfaces) were excluded. Screening and selection were in accordance with the Preferred Reporting Items for Systematic Reviews and Meta‐Analyses (PRISMA) guidelines (Moher et al. 2009) (Figure 1).

### 2.3 Data Extraction Using AI‐Assisted Workflow

For structured extraction of parameters of interest, we used the AI literature review platform Elicit (Elicit 2025). All included full‐text articles were uploaded to the platform, and structured queries were submitted to extract specific data fields, including study description, detailed preprocessing steps, and reported outcomes. These structured prompts are publicly accessible at https://github.com/neurogeriatricskiel/LitExtract.

### 2.4 Output Verification and Organization

The output from Elicit was initially extracted as raw CSV files. A thorough manual verification and cross‐referencing process was carried out against the original articles to ensure accuracy and completeness of the extracted data. This step produced a clean CSV file (available in the GitHub repository), which served as the finalized dataset for subsequent analysis.

### 2.5 Defining Preprocessing Steps

Preprocessing terminologies varied widely across studies and were therefore standardized into a defined vocabulary of keywords (Table 1). This schema represents a pragmatic first‐pass to enable cross‐study comparison. These were the terminologies agreed upon by three co‐authors, VV, LJP, and JW. Functionally equivalent terms (e.g., “high‐pass filter”, “remove drift”) were grouped under a common, standardized keyword label (e.g., “*highpass_filter*”). For clarity and consistency, subsequent keyword usages refer to the following definitions. The defined terms were further organized according to the sequential major pipeline stages: Raw data, Pre‐ICA Signal Cleaning, Pre‐ICA Data Preprocessing, ICA, and Post‐ICA. Table 1 summarizes the standard preprocessing steps, their representative terminology, and their definitions as referenced in this study. Because grouping choices and the stage architecture may influence transition frequencies, heterogeneity patterns should be interpreted with respect to the chosen ontology.

**TABLE 1:** Overview of preprocessing keywords and their definitions.

Stage of pipeline	Keyword	Definition/examples
Raw data	*Raw data*	Unprocessed EEG as recorded from the acquisition system
Pre‐ICA Signal Cleaning	*Channel removal*	Manual removal of noisy or disconnected channels based on impedance or variance
	*High‐pass filter*	Removal of slow drifts (e.g., < 0.5–1 Hz) using a high‐pass filter
	*Low‐pass filter*	Attenuation of high‐frequency activity above the cutoff (e.g., > 40–100 Hz)
	*Bandpass filter*	Retention of activity within a defined range (e.g., 1–50 Hz)
	*Notch filter*	Removal of narrowband noise, typical line noise at 50–60 Hz (e.g., CleanLine, bandstop)
	*Downsample*	Reduction of data sampling rate
Pre‐ICA Data Preprocessing	*Bad channel detection*	Identification of noisy channels (e.g., impedance, variance, kurtosis, or correlation‐based methods)
	*Artifact rejection*	Removal of signal segments with artifacts via manual inspection or automated algorithms (e.g., *iCanClean, clean_artifacts, clean_rawdata*, Principal Component Analysis [PCA], Canonical Correlation Analysis [CCA]) (detailed in Supporting Information Data S1)
	*Re‐reference*	Adjustment of reference (e.g., average reference, linked mastoids)
	*Epoching*	Segmentation of continuous EEG into time‐locked epochs
ICA	*IC decomposition*	Independent Component Analysis (ICA; e.g., AMICA, Infomax) to separate neural from artifactual sources
	*IC rejection*	Removal of artifactual components identified manually or via algorithms such as ICLabel or ADJUST
Post‐ICA	*Clustering*	Grouping ICs across subjects for meta‐analysis (e.g., k‐means)
	*Baseline correction*	Subtracting baseline activity relative to pre‐event interval
	*Dipole_fitting*	Source modeling of IC scalp maps using equivalent current dipoles
	*Normalization*	Scaling or z‐transforming data across participants or trials
	*Despiking*	Detection and correction of brief signal transients or spikes

### 2.6 Data Processing and Analysis

The finalized, manually verified dataset was further processed using a custom Python script (available in the GitHub repository). This script was used to restructure the cleaned .csv file by parsing multi‐entry fields, normalizing terminology, and aligning all extracted preprocessing information with the standardized keyword vocabulary and predefined pipeline stages (Table 1). To ensure consistency of the standardization process, the initial keyword assignments and complete preprocessing step extraction for all included studies were independently reviewed by two reviewers (LJP and VV). Discrepancies were resolved through discussion, ensuring that functionally equivalent preprocessing steps were consistently categorized across the dataset.

Specifically, reported preprocessing steps, artifact rejection methods, and outcome measures were split into individual entries, cleaned for inconsistencies, and mapped to their corresponding standardized labels to enable cross‐study aggregation and comparison. Based on this structured dataset, descriptive statistics were computed to summarize study characteristics, preprocessing step frequencies, and step‐to‐step transitions within pipelines. These summaries were used to quantify and visualize methodological diversity across studies.

## 3 Results

Sixty‐five studies met the predetermined inclusion criteria. These studies were characterized based on participant cohorts, gait tasks, EEG hardware, and preprocessing steps. The dataset consisted of diverse experimental designs and recording setups, reflecting the heterogeneity of gait‐related mobile EEG research.

### 3.1 Study Cohorts and Gait Tasks

**FIGURE 2:**
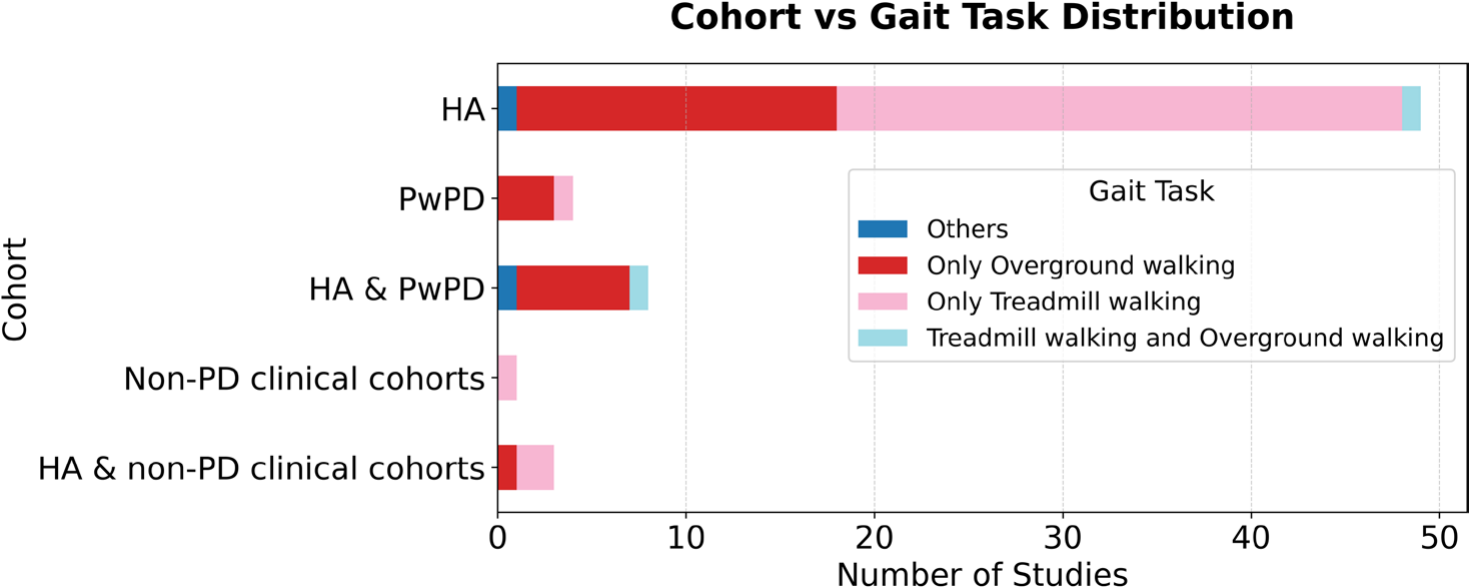
Distribution of studies by cohort and gait task. The horizontally stacked bars indicate the number of studies investigating each gait task within specific participant cohorts. HA—Healthy adults; PwPD—People with Parkinson's disease; non‐PD clinical cohorts—Stroke survivors, amputees, spinal cord injured cohorts; Other gait task—Stepping‐in‐place.

The majority of the included gait‐related mobile EEG studies investigated healthy adults (76.1%), followed by people with Parkinson's disease (PwPD; 17.9%). Non‐PD clinical cohorts (6.0%) were spinal cord injury patients, stroke survivors, and amputees. Treadmill walking was the most commonly investigated task (52.3%), followed by overground walking (41.5%) and stepping‐in‐place tasks (3.1%). Notably, only two studies (3.1%) employed both treadmill and overground conditions within the same experiment. When stratified by cohort, treadmill walking was the most common paradigm among healthy adults (58.8%), while overground walking was done more in studies involving PwPD (75.0%). Figure 2 shows the distribution of cohorts across gait paradigms.

### 3.2 EEG Hardware and Gait Measurement Setups

**FIGURE 3:**
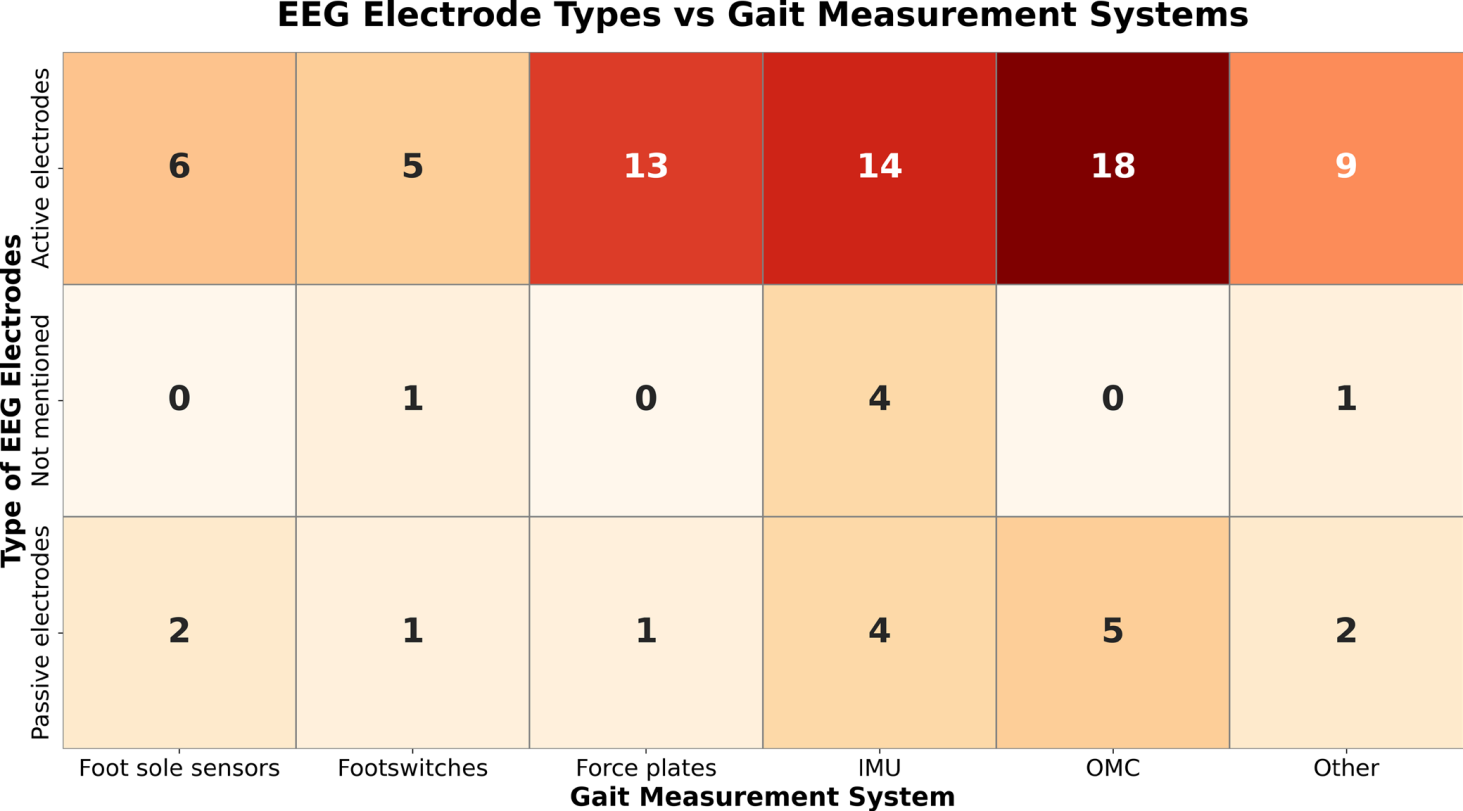
Heatmap of EEG electrode types and gait measurement setups. The color intensity of each cell reflects the frequency of co‐occurrence between the specific EEG electrode type and the gait setup in the included studies. IMU—Inertial Measurement Unit; OMC—Optical Motion Capture; Other—GAITrite electronic walkway, virtual environment tracking, OptoGait system.

To further characterize diversity in EEG and gait hardware used, we mapped the relationship between the types of EEG electrodes and the gait measurement setups used. Active EEG electrodes dominated the studies (75.6%), with passive electrodes in 17.4%; the remaining 7% did not report electrode type. Among gait measurement systems, optical motion capture (OMC; 26.7%) and inertial measurement units (IMU; 25.6%) were most common, followed by force plates (16.3%), other systems (14%), such as electronic walkways and virtual environment tracking. The combination patterns reflected these preferences. Active electrodes were most commonly paired with OMC (27.7%) and IMUs (21.5%), with smaller proportions using force plates (20%) or “other” systems (13.8%). Passive‐electrode studies tended to rely on OMC (33.3%) or IMUs (26.7%). Figure 3 summarizes these co‐occurrence patterns, highlighting which EEG‐gait hardware configurations appear most consistently across the literature and which remain comparatively rare.

### 3.3 Overview of Preprocessing Steps

The included studies demonstrated wide variation in how mobile EEG data were cleaned and prepared. Across all 65 studies, 18 distinct preprocessing steps were reported, and their usage frequencies differed substantially. Artifact rejection was the most common step, appearing in 102 instances across 64 out of 65 included studies, followed by ICA decomposition (87.7%), re‐referencing (83.1%), and IC rejection (78.5%). Filtering approaches varied more strongly: 53.8% applied a high‐pass or band‐pass filter, 35.4% used a notch filter, and 32.3% used low‐pass filtering.

**FIGURE 4:**
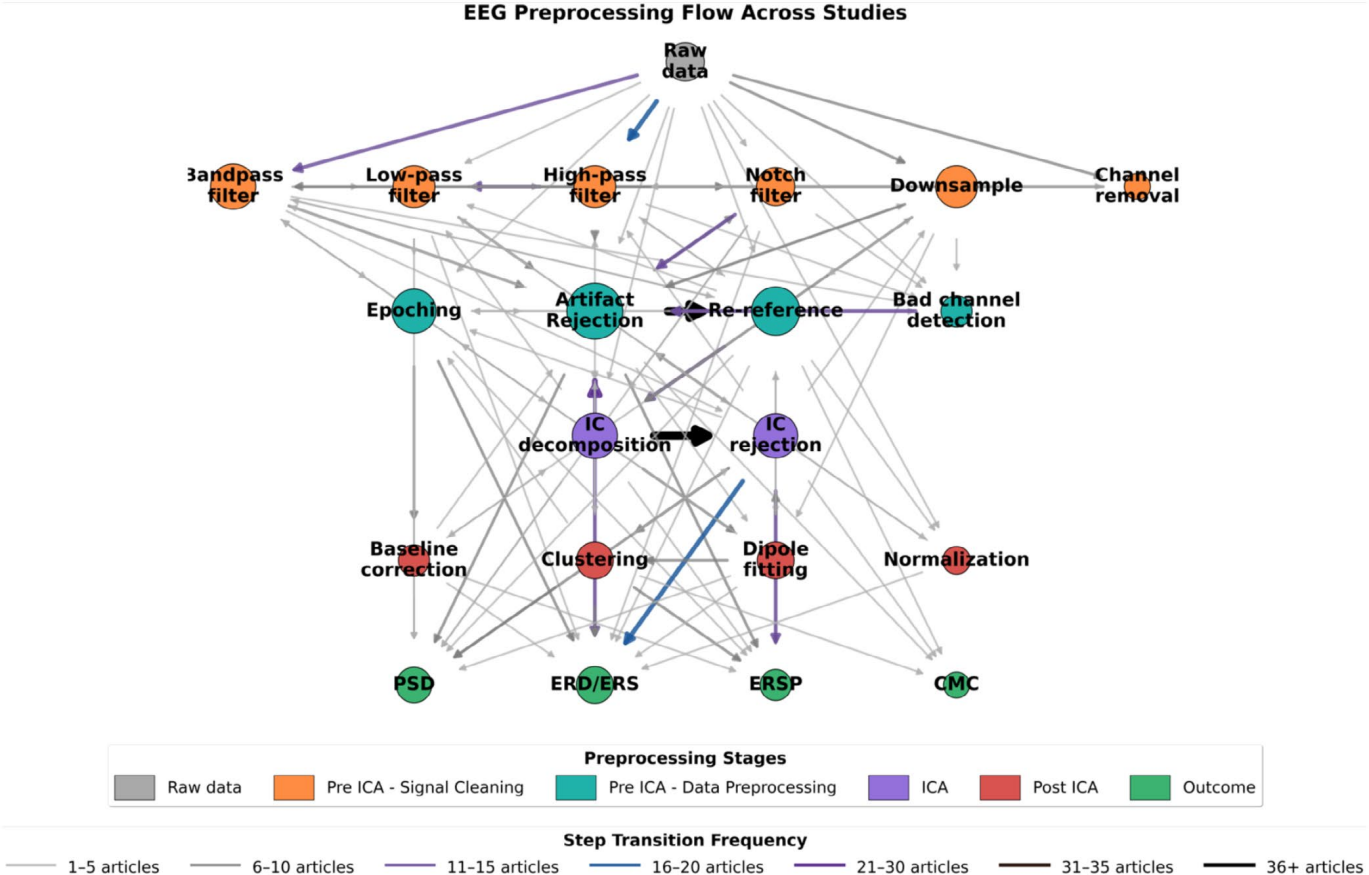
Flow of different preprocessing pipelines across multiple studies. The nodes represent standardized preprocessing steps, grouped by stage (Raw data, Pre‐ICA Signal Preservation, Pre‐ICA Preprocessing, ICA, Post‐ICA Processing and Outcomes). Node sizes indicate the number of studies using a certain step. Arrows denote the transition from one step to another, with the color and thickness indicating the frequency of that transition.

Variation was also evident in how steps were combined. The most frequent transitions across all pipelines were IC decomposition to IC rejection (5.9%) and artifact rejection to re‐referencing (5.9%), reflecting a widely adopted ICA‐based cleaning strategy. Other common transitions included artifact rejection to IC decomposition (3.8%), raw data to high‐pass or band‐pass filtering (2.8%–2.4%). In the downstream, IC rejection often led to outcome‐specific steps such as ERD/ERS (3%) or ERSP (1.9%), indicating that post‐ICA processing steps were limited (Figure 4).

To further explore where the choice of preprocessing step varied with the outcomes, we examined pipelines separately for the outcomes outlined here—PSD, ERSP, ERD/ERS, and CMC (see Figures B[i]–[iv] in Data S2).

### 3.4 Diversity in Artifact Rejection Methods

**FIGURE 5:**
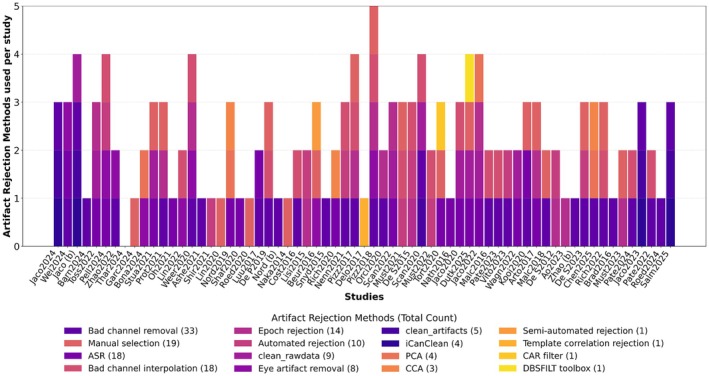
Artifact rejection methods used across all included studies. Each bar represents a study, with stacked segments indicating the artifact rejection techniques reported. The legend shows the frequency of each method across the full dataset. ASR—Artifact Subspace Reconstruction; CCA—Canonical Correlation Analysis; PCA—Principal Component Analysis.

To characterize how studies operationalized the broad “artifact rejection” step, we quantified the frequency of methods used (Figure 5). Among the 65 studies implementing artifact rejection, we identified 16 distinct techniques. The most common approaches were bad channel removal (50.8%), followed by manual selection of noisy channels (29.2%), ASR (27.7%), and bad channel interpolation (27.7%). Additional approaches included epoch rejection (21.5%), automated rejection algorithms (15.4%), *clean_rawdata* (13.8%), eye‐artifact removal (12.3%), and *iCanClean* algorithm (6.2%).

The number of artifact rejection techniques used per study ranged from 1 to 5, with a mean of 2.29 (SD = 1.18). Forty‐seven studies (72.3%) combined multiple methods, highlighting the use of hybrid pipelines with manual and automated strategies to mitigate gait‐related artifacts.

## 4 Discussion

This review maps the methodological landscape of gait‐related mobile EEG research and reveals substantial heterogeneity in preprocessing pipelines across tasks, cohorts, and hardware configurations. Such diversity is not inherently problematic. Mobile EEG spans a wide range of tasks, from controlled treadmill walking to unconstrained overground locomotion, each producing distinct noise signatures. The challenge, therefore, is not eliminating variability but making it interpretable and transparent with regard to study context and analytical goals.

A recurring gap in current mobile EEG literature is the context of “mobility”. Mobility in mobile EEG is task‐dependent and can vary with context. It can be characterized by device mobility (e.g., tethered vs. untethered) and participant mobility (e.g., controlled treadmill vs. unconstrained overground walking). These differing mobility paradigms produce distinct noise profiles. Controlled or constrained treadmill paradigms tend to produce gait‐synchronous artifacts, whereas free overground walking generates transient, high‐amplitude bursts from contact and cable movement (Gorjan et al. 2022; Ledwidge et al. 2025; Nathan et al. 2016). These differences matter because preprocessing choices should be driven by the specific noise profile of the recording and not by convention. Frameworks such as Bateson's CoME scoring system illustrate how a quantified mobility metric could guide “fit‐for‐purpose” preprocessing (Bateson et al. 2017).

Apart from the different nature of noise, our review highlights that artifact rejection remains one of the most variable and unevenly reported steps across the included studies. Automated EEGLAB plug‐ins, such as *clean_rawdata* and iCanClean, are implemented for artifact rejection (Figure 5). These plug‐ins contain multiple functionalities (e.g., high‐pass filtering, ASR) and are most often not specified in studies. Without specifying which functionalities were enabled and which parameter values were used, two pipelines that both use “*clean_rawdata*” may be performing different operations. This opacity limits comparability more than the choice of algorithm itself. The same holds for ICA, wherein implementations may differ in filter settings, component‐pruning criteria, and dipole‐fitting thresholds (Artoni et al. 2025), yet these details are not frequently reported in manuscripts. Importantly, the suitability of these preprocessing decisions depends on the downstream outcome measure, since ERSP, ERD/ERS, PSD, and CMC differ in their sensitivity to filtering, temporal alignment, channel rejection, and component selection.

Another preprocessing step, which showed large heterogeneity in implementation, was the identification and removal of “bad” channels. It has been established that, for example, head acceleration and the EEG amplitude can be correlated in EEG channels (Kline et al. 2015). Channel deviations can also arise from cable movements, which are common in mobile recordings. However, there does not seem to be a standard or common practice for how bad channels are identified. Most commonly, visual inspection, channel interpolation, and channel removal based on statistical criteria have been reported. Oliveira and colleagues proposed to use a template correlation approach (Oliveira et al. 2017). They demonstrated that retained artifact channels had ∼60% higher delta power and showed ERSP patterns locked to walking, underscoring why channel removal is essential for reliable ERSP/ERD and CMC analyses.

A fundamental challenge is the lack of ground truth for evaluating ICA decompositions in real mobile EEG data, making it impossible to determine whether neural and artifactual sources have been correctly separated. ICA performance depends on how well the data meet assumptions such as source independence and relative signal stationarity. Violations of these assumptions are not unique to mobile EEG and can also occur in stationary laboratory recordings, depending on acquisition settings and preprocessing choices. However, gait‐related recordings introduce additional movement‐related activity that can further complicate source separation and component interpretation. Snyder and colleagues (Snyder et al. 2015) demonstrated that ICA with dipole fitting can produce components that appear cortical but are purely artifactual when applied to gait‐related movement artifact data. They note that temporally correlated artifact sources, such as semi‐periodic gait‐locked signals, can lead to spatial superposition during decomposition. Another study (Kline et al. 2015) complements this by showing that movement artifact varies across electrodes and walking speed in ways that simple removal methods cannot fully address. ICA performance also strongly depends on preprocessing choices applied before decomposition, in particular the high‐pass filter cutoff, which directly affects decomposition quality (Klug et al. 2021; Winkler et al. 2015). Finally, component classification varied considerably across the reviewed studies; manual inspection, ICLabel, and ADJUST can yield different rejection sets on the same data, yet the classifier, its thresholds, and the number of retained components were inconsistently reported. These limitations do not argue against ICA, but highlight that its effectiveness in mobile EEG is conditional on preprocessing decisions that require transparent documentation. Additionally, studies (N. S. J. Jacobsen et al. 2022; Seeber et al. 2015) showed that adding spectral PCA before ICA decomposition might additionally help attenuate artifacts, an approach that has been reported in only 4 out of 65 studies in this review.

Baseline correction was rare in the included studies, with only 10.4% applying it for ERSP/ERD. For gait‐aligned data, researchers often subtract the average power of each gait cycle or use a preceding‐cycle window (Herbert et al. 2020; N. S. J. Jacobsen et al. 2022). Alternatively, baselines can be taken from quiet standing or the entire dataset (condition average) (C. Liu et al. 2024). It is important to acknowledge the impact of the selection of a suitable baseline on the outcomes. However, this relationship has not been systematically explored in the context of mobile gait‐related EEG activity (Alday 2019).

The term “under‐reporting” mentioned in this review not only corresponds to individual parameters but also to a wide spectrum of reporting practices in general. A few studies provide full preprocessing scripts, versioned repositories, and parameter‐level details but describe their preprocessing in vague terms, offering minimal insight into the parameters used. This variability in reporting and inconsistent use of preprocessing terminologies presents a barrier to transparency. Principles from research‐software transparency, such as the FAIR‐R guidelines (Barker et al. 2022), emphasize the need for documentation so that one can find it, access it, and implement it. In practice, this means sharing code or workflows via open‐source repositories and using the manuscript to explain key decisions (Welzel et al. 2025).

The predominance of healthy adult cohorts implies that pipelines are usually established under controlled conditions before moving to more complex clinical populations. As hardware and multimodal synchronization improve, the gradual shift toward clinical applications presents an opportunity to introduce reporting standards. Without consistent documentation, differences in preprocessing will make cross‐study comparisons harder just as the field becomes more clinically relevant.

These reporting gaps matter because different preprocessing steps can strongly affect the four above‐mentioned outcomes. PSD shows good retest reliability (Popov et al. 2023), but ERSP and ERD/ERS depend on accurate gait alignment and can change noticeably with different filters or channel decisions (Bonassi et al. 2024; Gwin et al. 2011; Oliveira et al. 2017). CMC is even more sensitive, since both EEG and EMG need to be clean for reliable coherence estimates (Liu et al. 2019). As a result, two studies may report different findings simply because of differences in artifact handling, ICA settings, or baseline choice, not because the underlying brain activity truly differs.

The interaction between EEG preprocessing and other measurement setups reinforces this point. For example, two treadmill‐walking studies may differ only in whether gait events are derived from IMUs or reflective markers. The EEG preprocessing pipeline itself may not change, but the precision of event timing and segmentation will affect outcomes such as ERSP or ERD/ERS when EEG methods are identical. This reinforces that methodological transparency must extend beyond EEG preprocessing to include EEG preprocessing and gait‐synchronization procedures.

To summarize, gait‐related mobile EEG does not require a single standardized pipeline, but it does require standardized reporting. Clear descriptions of context, artifact rejection choices, and software parameters would help ensure methodological diversity supports the field rather than making results difficult to compare.

## 5 Conclusion

Methodological diversity in gait‐related mobile EEG reflects a rapidly evolving field adapting to different hardware configurations, mobility contexts, and analytical goals. However, to contribute to cumulative progress, transparency is important. Our review highlights the reporting gaps that challenge the reproducibility of findings. Rather than prescribing a single optimal workflow, this review provides a structured overview of current preprocessing practices and their variability across gait‐related mobile EEG studies. By adopting clearer documentation practices, from standardized reporting of key parameters to making preprocessing scripts accessible, the field can maintain its methodological flexibility while ensuring scientific accountability.

## 6 Future Outlook

Advances in gait mobile EEG require a progression from individual best practices to shared community guidelines. A practical next step is adopting a minimal set of reporting items that cover key preprocessing decisions: filtering parameters, artifact‐rejection criteria, ICA settings, baseline correction, gait‐event detection methods, and software plug‐in uses. Even this basic level of transparency would allow researchers to understand how different pipelines shape the four major outcomes: PSD, ERSP, ERD/ERS, and CMC.

Further, community efforts could build this foundation by curating benchmark datasets with quantified mobility scores, such as those defined by the CoME framework. Access to open‐source datasets would allow researchers to compare pipelines under controlled conditions, evaluate how specific steps influence neural outcomes, and test how well methods generalize across mobility contexts and populations.

Developing shared evaluation metrics is another important direction. For example, metrics that quantify the preservation of neural structure, suppression of motion‐related noise, or stability of gait‐locked features would make it easier to compare methods objectively rather than relying on qualitative judgment.

Lastly, transparent software practices will become increasingly important. Publishing code, workflows, and project repositories aligns with standards in research software (e.g., FAIR‐R) and ensures that preprocessing pipelines are both interpretable and replicable. We want to stress that standardization is not a constraint on methodological innovation but rather a foundation for establishing a framework of standards that the community can agree on.

## Author Contributions

**Lara Johanna Papin:** data curation, validation, writing – review and editing. **Robbin Romijnders:** conceptualization, writing – review and editing. **Vaishali Vinod:** conceptualization, data curation, methodology, software, validation, visualization, writing – original draft. **Walter Maetzler:** supervision, writing – review and editing. **Julius Welzel:** conceptualization, software, supervision, validation, visualization, writing – review and editing.

## Funding

This work was supported by Deutsche Forschungsgemeinschaft, 464552782.

## Conflicts of Interest

The authors declare no conflicts of interest.

## Supporting information


**Data S1:** Artifact rejection methods used in gait‐related mobile EEG studies.

**Data S2:** Outcome‐specific preprocessing pipelines used in Gait‐related Mobile EEG studies included in the review.

**Data S3:** Common combinations of preprocessing steps in gait‐related mobile EEG studies.

## Data Availability

The data that support the findings of this study are openly available in LitExtract at https://github.com/neurogeriatricskiel/LitExtract.
