# Supplementary material for: Preprocessing on the Go: Practices in Gait‐Related Mobile EEG
Source: Psychophysiology. 2026 Jun 25;63(6):e70352. doi: 10.1111/psyp.70352 (PMC13296838; doi:10.1111/psyp.70352)
Supplement: Supplementary file 1 — Data S1: Artifact rejection methods used in gait‐related mobile EEG studies. [file PSYP-63-e70352-s002.docx]

**Artifact Rejection Methods used in Gait-Related Mobile EEG Studies**

Table A. Overview of artifact rejection techniques reported across included studies, with descriptions

| **Artifact rejection methods** | **Description** |
| --- | --- |
| Bad channel removal | Excludes electrodes with poor correlation, flat signals, or excessive noise to prevent contamination in later stages |
| Manual selection | Visual inspection and exclusion of bad channels or segments |
| ASR | Artifact Subspace Reconstruction: Detects and surpasses transient, high-amplitude artifacts using subspace reconstruction over time windows. |
| Bad channel interpolation | Reconstructs removed or noisy channels from neighboring electrodes to preserve spatial information. |
| Epoch rejection | Removes noisy time segments exceeding amplitude or statistical thresholds to improve data reliability. |
| Automated rejection | Algorithmic detection and exclusion of artifacts using amplitude, kurtosis, or probability criteria. |
| *clean_rawdata* | Multifunctional EEGLAB plug-in combining ASR, bad-channel detection, and interpolation |
| Eye artifact removal | Removes blink and saccade artifacts via ICA or regression while retaining frontal neural activity. |
| *clean_artifacts* | EEGLAB plug-in performing automated detection and removal of high-amplitude transients and noisy channels. |
| *iCanClean* | Automated CCA-based cleaning separates motion and muscle noise from neural signals; improves data quality before ICA. |
| PCA | Principal Component Analysis; Reduces data dimensionality or isolates dominant noise sources before ICA. |
| CCA | Canonical Correlation Analysis: Identifies correlated non-neural noise across channels or modalities for targeted artifact removal. |
| Semiautomated rejection | Combines automated artifact detection with manual verification for balanced accuracy and control. |
| Template correlation rejection | Rejects segments that match predefined artifact templates (e.g., blinks, EMG bursts). |
| CAR filter | Common Average Reference: Re-references each channel to the average of all channels, reducing spatially widespread noise. |
| DBSFILT toolbox | Specialized toolbox for filtering and removing artifacts related to deep brain stimulation or similar sources. |
