# Supplementary material for: Preprocessing on the Go: Practices in Gait‐Related Mobile EEG
Source: Psychophysiology. 2026 Jun 25;63(6):e70352. doi: 10.1111/psyp.70352 (PMC13296838; doi:10.1111/psyp.70352)
Supplement: Supplementary file 2 — Data S2: Outcome‐specific preprocessing pipelines used in Gait‐related Mobile EEG studies included in the review. [file PSYP-63-e70352-s001.docx]

**Outcome-specific preprocessing pipelines used in Gait-related Mobile EEG studies included in the review**


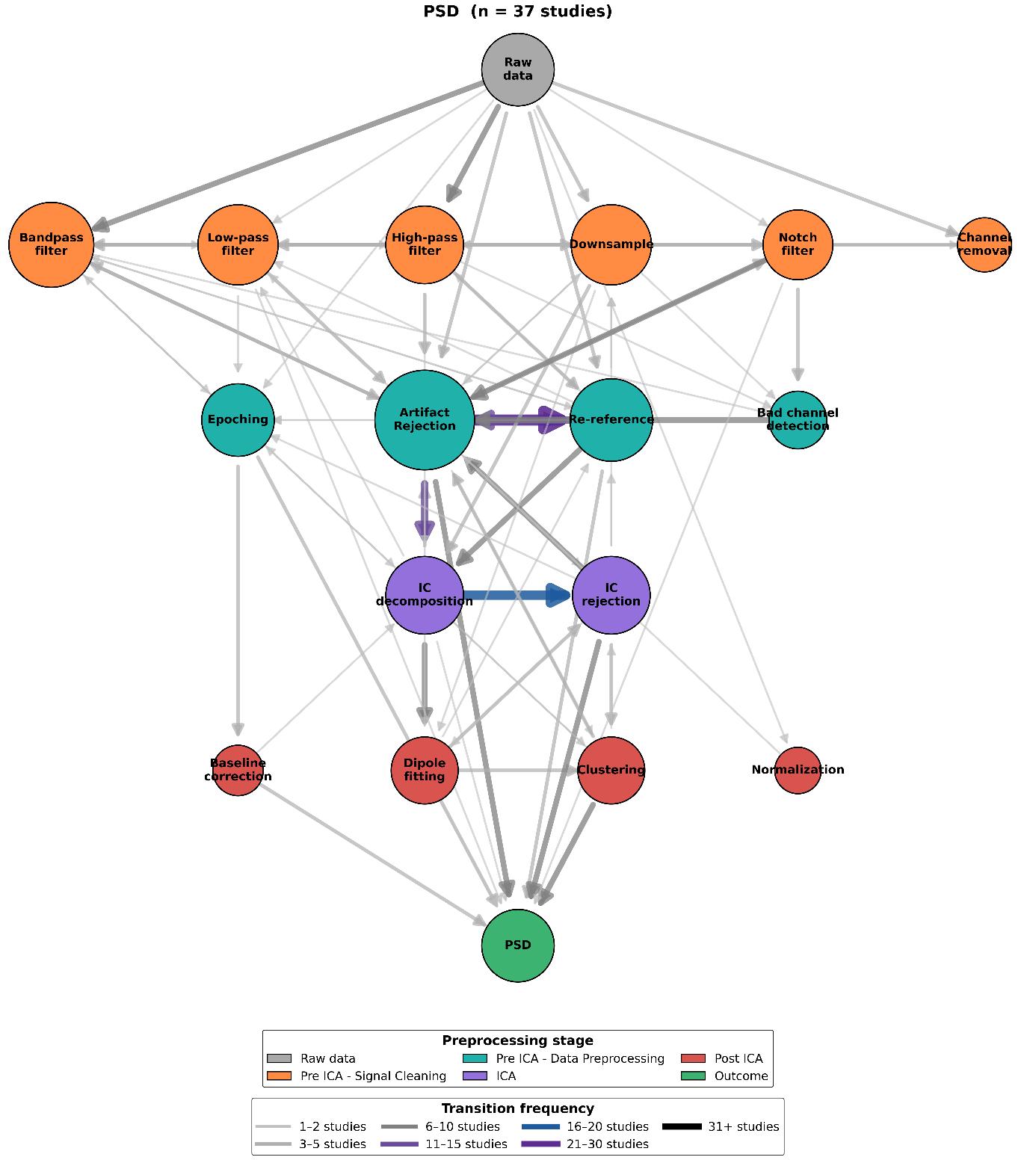


*Fig.B(i). Preprocessing pipelines for studies reporting Power Spectral Density (PSD). The nodes represent standardized preprocessing steps, grouped by stage (Raw data, Pre-ICA Signal Preservation, Pre-ICA Preprocessing, ICA, Post-ICA Processing and Outcomes). Node sizes indicate the number of studies using a certain step. Arrows denote the transition from one step to another, with the color and thickness indicating the frequency of that transition.*


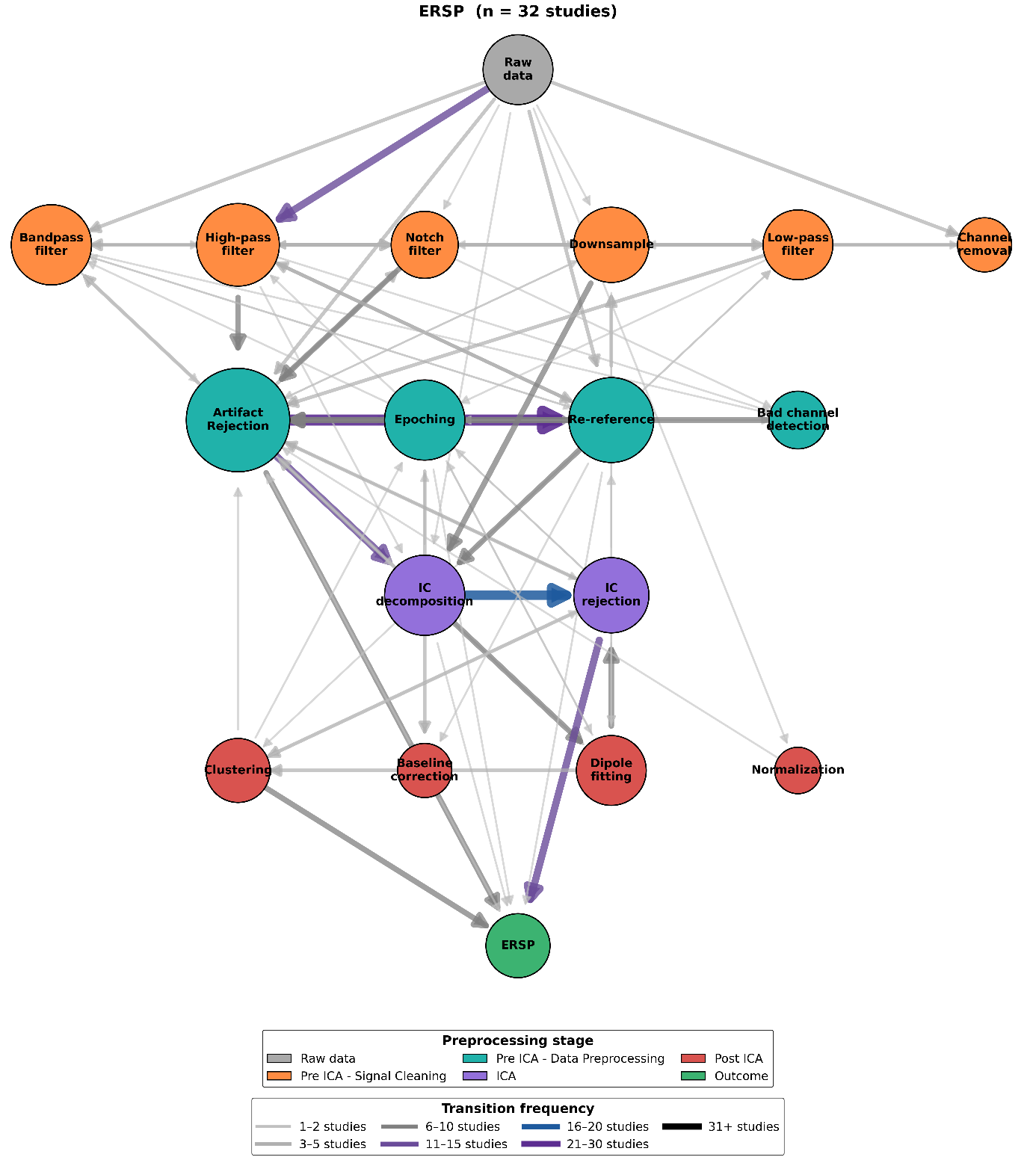


*Fig.B(ii). Preprocessing pipelines for studies reporting Event-Related Spectral Perturbation (ERSP). The nodes represent standardized preprocessing steps, grouped by stage (Raw data, Pre-ICA Signal Preservation, Pre-ICA Preprocessing, ICA, Post-ICA Processing and Outcomes). Node sizes indicate the number of studies using a certain step. Arrows denote the transition from one step to another, with the color and thickness indicating the frequency of that transition.*


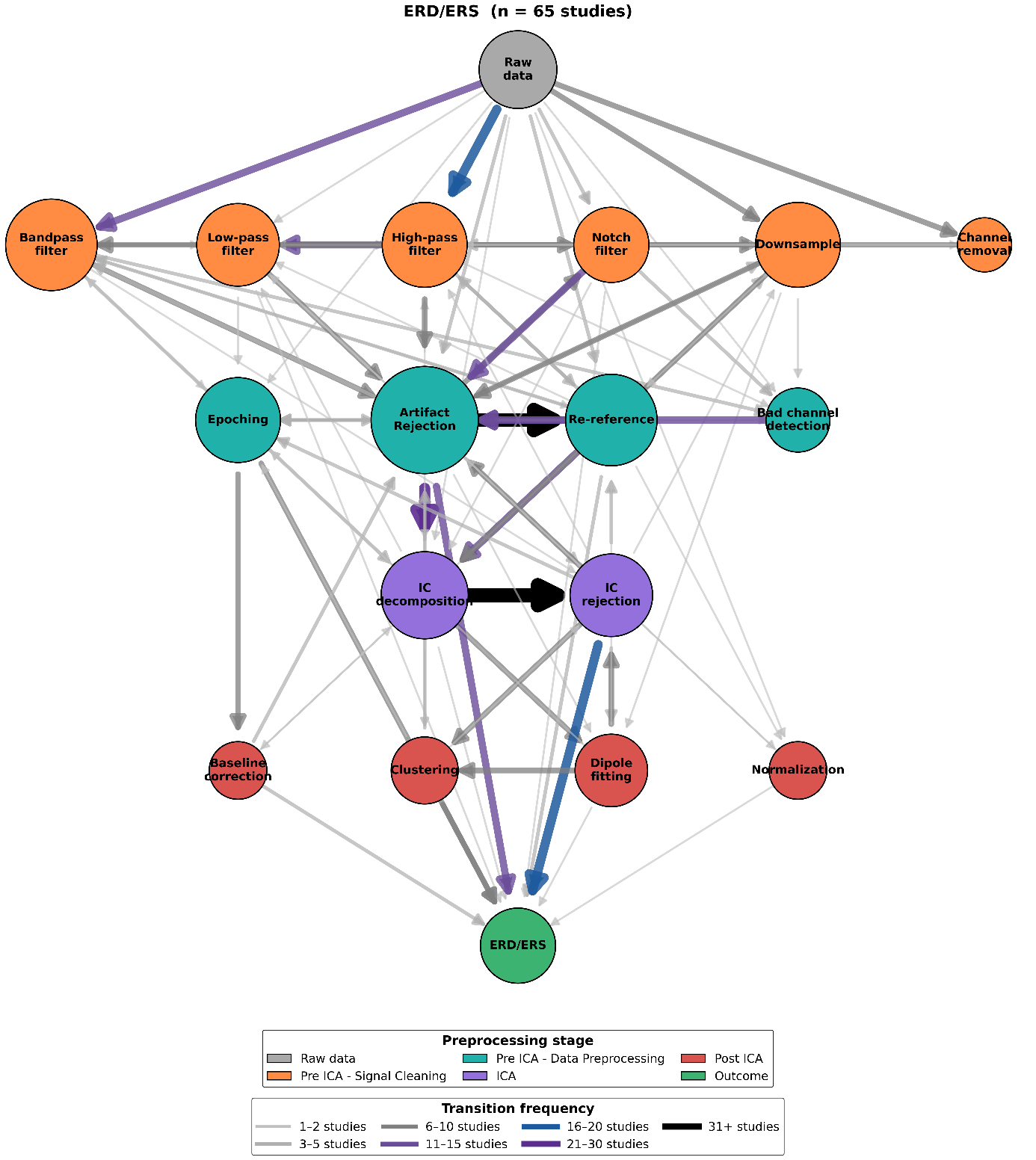


*Fig.B(iii). Preprocessing pipelines for studies reporting Event-related (de-)synchronization (ERD/ERS). The nodes represent standardized preprocessing steps, grouped by stage (Raw data, Pre-ICA Signal Preservation, Pre-ICA Preprocessing, ICA, Post-ICA Processing and Outcomes). Node sizes indicate the number of studies using a certain step. Arrows denote the transition from one step to another, with the color and thickness indicating the frequency of that transition.*


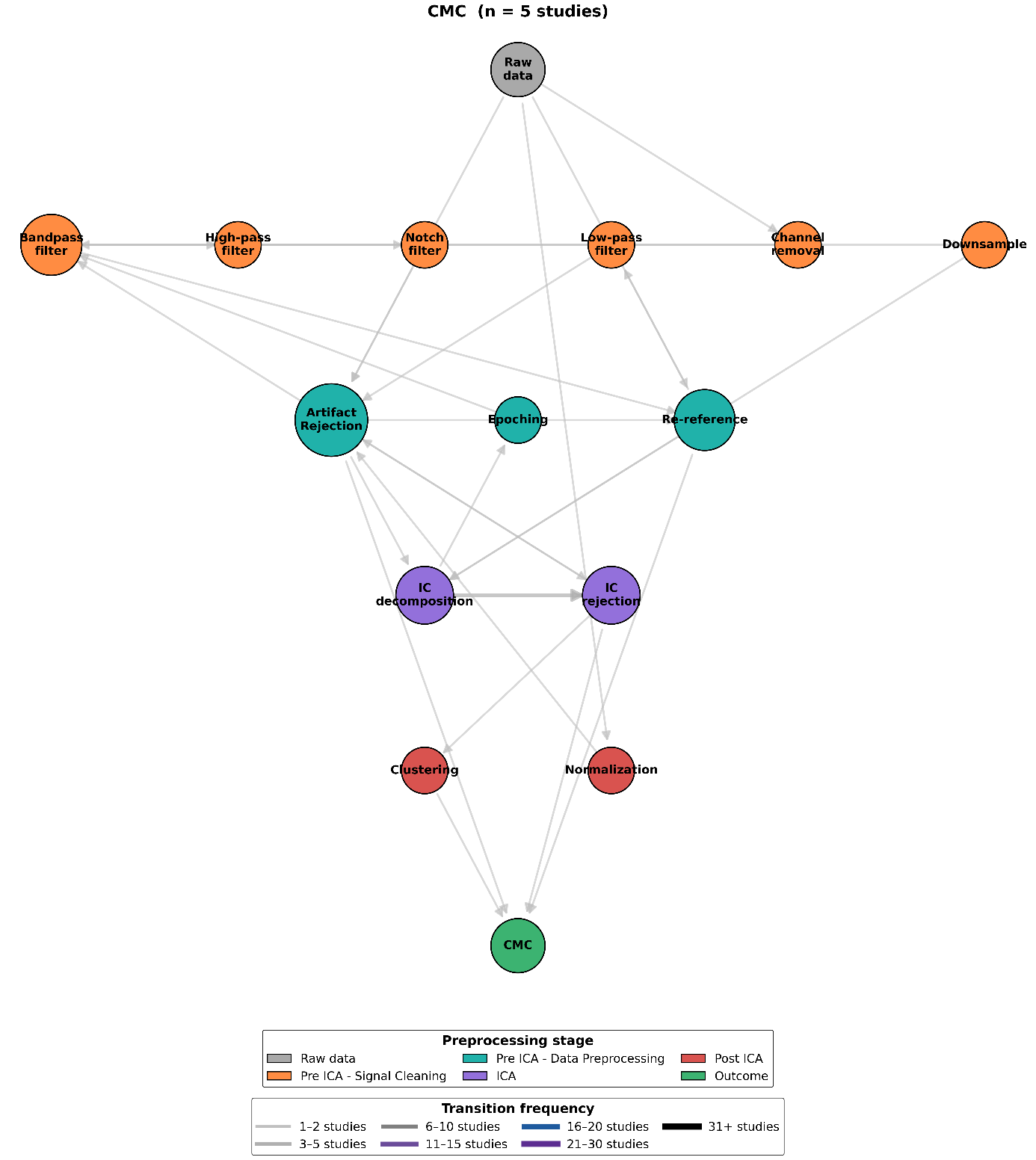


*Fig.B(iv). Preprocessing pipelines for studies reporting Corticomuscular Coherence (CMC). The nodes represent standardized preprocessing steps, grouped by stage (Raw data, Pre-ICA Signal Preservation, Pre-ICA Preprocessing, ICA, Post-ICA Processing and Outcomes). Node sizes indicate the number of studies using a certain step. Arrows denote the transition from one step to another, with the color and thickness indicating the frequency of that transition.*
