# Supplementary material for: Preprocessing on the Go: Practices in Gait‐Related Mobile EEG
Source: Psychophysiology. 2026 Jun 25;63(6):e70352. doi: 10.1111/psyp.70352 (PMC13296838; doi:10.1111/psyp.70352)
Supplement: Supplementary file 3 — Data S3: Common combinations of preprocessing steps in gait‐related mobile EEG studies. [file PSYP-63-e70352-s003.docx]

**Common Combinations of Preprocessing Steps in Gait-Related Mobile EEG Studies**


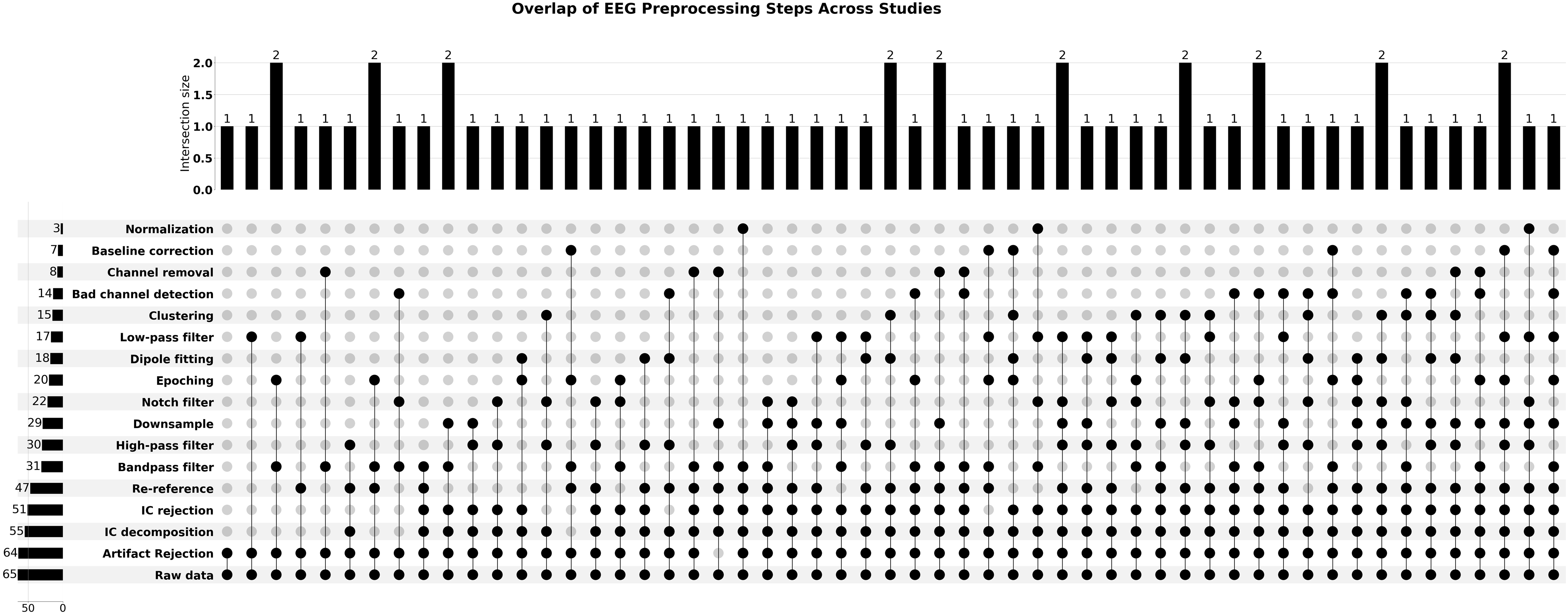


Figure A. UpSet plot showing intersections of preprocessing steps across 65 studies. The vertical bars indicate the number of studies implementing each unique combination, and the matrix below shows the constituent steps. Artifact rejection (n = 66), ICA decomposition (n = 57), and ICA component rejection (n = 53) were most frequent, with filtering, downsampling, and re-referencing also common. Artifact rejection and ICA decomposition co-occurred most strongly (n = 56), highlighting recurring pipeline cores while the plot also visualizes less frequent, diverse step combinations.
